# City-scale energetics: window on adaptive thermal insulation in North American cities

**DOI:** 10.1007/s00360-021-01411-8

**Published:** 2021-10-22

**Authors:** Richard W. Hill, Maxwell Grezlik, Timothy E. Muhich, Murray M. Humphries

**Affiliations:** 1 Department of Integrative Biology, Michigan State University, East Lansing, MI 48824 USA; 2 Department of Natural Resource Sciences, McGill University, Sainte-Anne-de-Bellevue, Quebec H9X 3V9 Canada; 3 Present Address: School for Marine Science and Technology, University of Massachusetts—Dartmouth, New Bedford, MA 02744 USA; 4 Present Address: Battle Creek Area Mathematics and Science Center, Battle Creek, MI 49017 USA

**Keywords:** Homeothermy, Energy conservation, Energy efficiency, Thermoregulation, Urban energy use, Built environment

## Abstract

Previous research demonstrated that cities are similar to individual mammals in their relationship between the rate of energy use for heating and outdoor air temperature (*T*_a_). At *T*_a_s requiring heating of indoor living spaces, the energy-*T*_a_ plot of a city contains information on city-wide thermal insulation (*I*), making it possible to quantify city-wide *I* by use of the city as the unit of measure. We develop methods for extracting this insulation information, deriving the methods from prior research on mammals. Using these methods, we address the question: in North America, are cities built in particularly cold locations constructed in ways that provide greater thermal insulation than ones built in thermally more moderate locations? Using data for 42 small and medium-size cities and two information-extraction methods, we find that there is a statistically significant inverse relationship between city-wide *I* and *T*_10-year_, the average city *T*_a_ over a recent 10-year period (range of *T*_10-year_: − 11 to 26 °C). This relationship represents an energy-conserving trend, indicating that cities in cold climates have greater built-in thermal insulation than cities in warm climates. However, the augmentation of insulation in cold climates is only about half as great as would be required to offset fully the increased energy cost of low *T*_a_s in a cold climate, and *T*_10-year_ explains just 5–11% of the variance in measured insulation, suggesting that cities in North America vary greatly in the extent to which thermal insulation has been a priority in city development.

**Supplementary Information:**

The online version contains supplementary material available at 10.1007/s00360-021-01411-8.

## Introduction

For analysis of continent-scale human energy use, a key question is whether people build better-insulated cities in places where superior thermal insulation would have the greatest effect. We argue in this paper that, for quantifying city thermal insulation, it may be possible to treat the city as the unit of measure by applying methods developed for the analysis of insulation in animal homeotherms. Our specific interest is in insulation as a defense against heat loss in cold environments.

At present, there is great multidisciplinary interest in the thermal insulation of the built environment in cities. Cities are where much of human energy use takes place (Burnside et al. 2012; Burger et al. 2017), and in the contemporary quest for improved energy efficiency to mitigate global warming, the thermal insulation provided by the urban built environment in cold climates is a pivotal consideration (Wilkinson et al. 2007; Rickwood et al. 2008). Recognizing the current multidisciplinary interest in insulation, we use this Introduction to explain our concepts for measuring city insulation in terms accessible to a multidisciplinary audience.

**Fig. 1:**
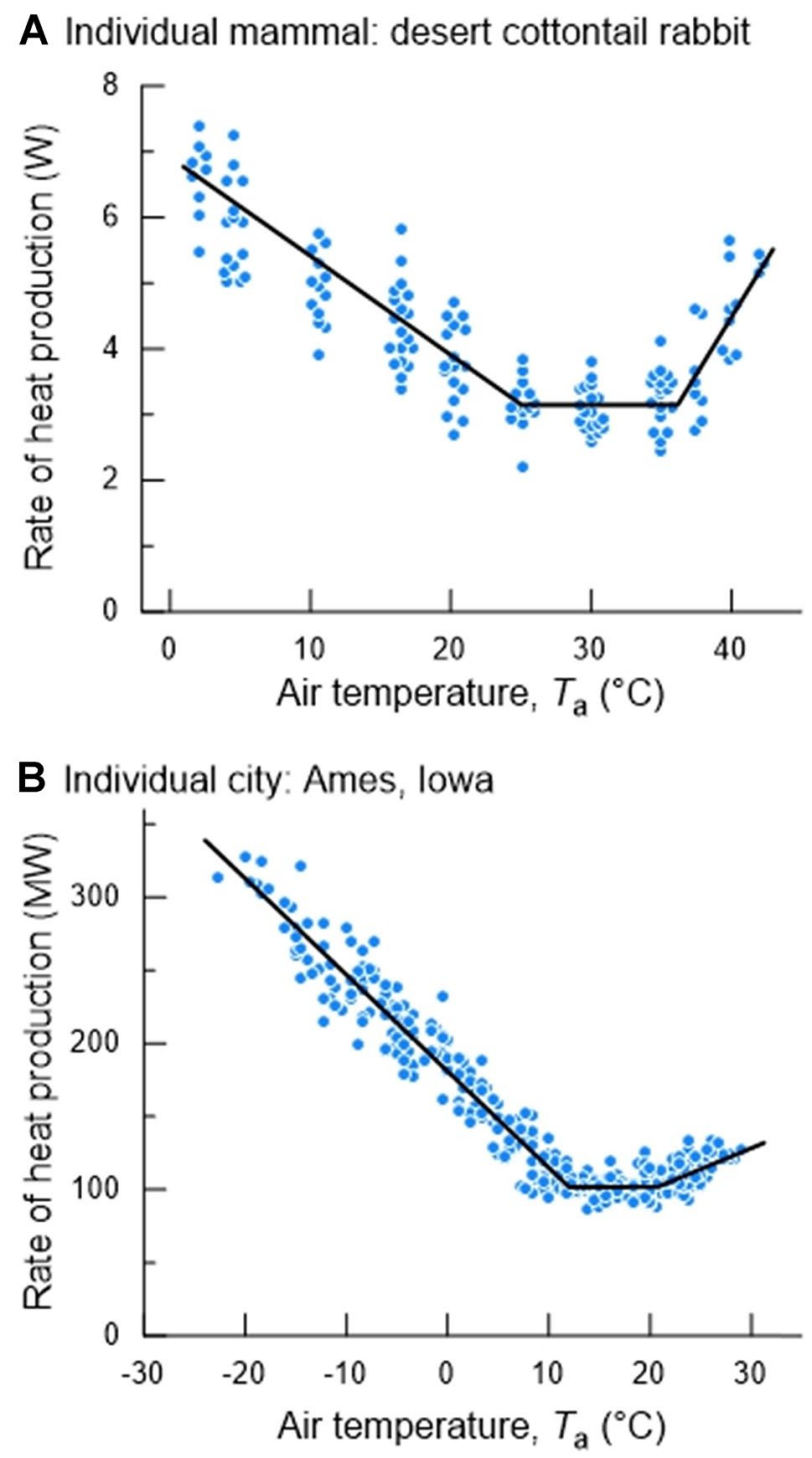
Rate of heat production as a function of air temperature (*T*_a_) in a mammal and a city. **A** Total individual metabolic rate as a function of *T*_a_ in winter-acclimatized rabbits, *Sylvilagus audubonii*. Each symbol represents one individual at one *T*_a_. **B** Total city rate of heat production by use of electricity and natural gas as a function of outdoor *T*_a_ in Ames, Iowa, in 2009. Each symbol represents one calendar day [**A** from (Hinds 1973); **B** from (Hill et al. 2013)]

Hill et al. (2013) demonstrated that individual cities behave like thermoregulating superorganisms: the total rate of energy use by the buildings in a city varies with daily outdoor air temperature (*T*_a_) in much the same way as the metabolic rate of a homeothermic animal varies with *T*_a_ (Fig. 1). As a shorthand expression, we will often call the relationship between the rate of heat production and *T*_a_ the “energy-*T*_a_” curve. In both cities and homeothermic animals, the curve has a similar shape because of similar underlying mechanisms, and because of this similarity, the extensive body of theory developed to understand the energetics of animal homeotherms can in principle be applied to understanding city energetics.

The thermal insulation, *I*, provided by the exterior construction of an individual building is a major determinant of the energy cost of heating the living space inside the building in winter. An entire city can be considered to have a city-wide value of *I*, which is a function of the values of *I* for the hundreds or thousands of individual buildings in the city. To estimate city-wide *I*, one approach would be reductionistic: treat individual buildings (or other living units) as the units of measure, estimate *I* for each, and synthesize. Such an approach would be fraught with privacy concerns and expensive. An alternative approach—far less fraught and expensive—is to treat the entire city as the unit of measure and calculate city-wide *I* from the city energy-*T*_a_ curve.

**Fig. 2:**
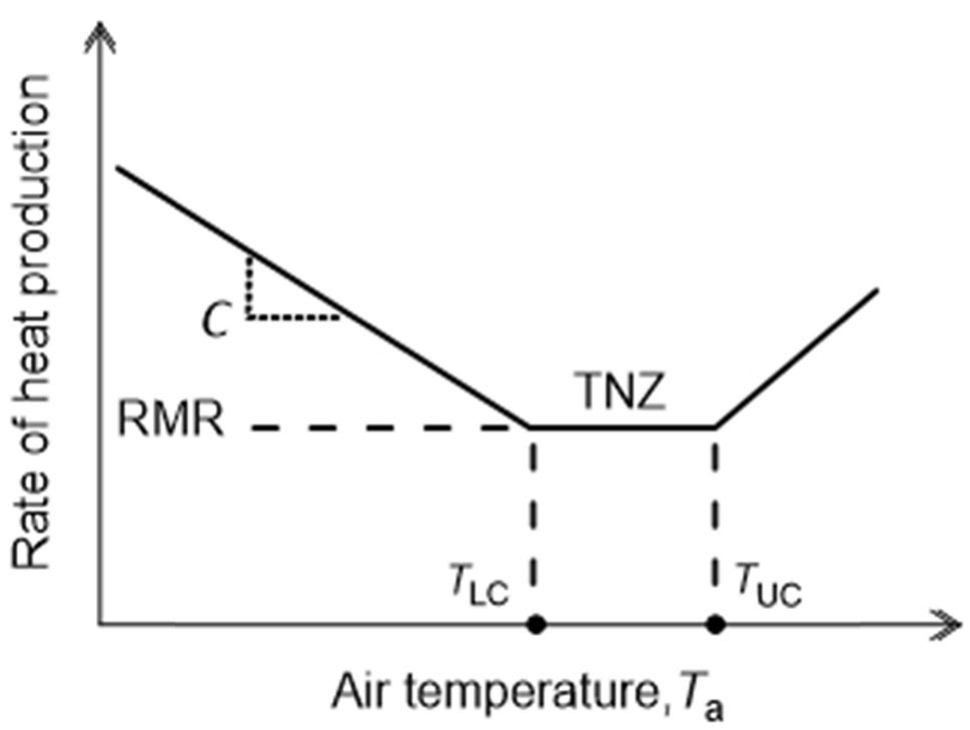
Standard model of the relation between the rate of heat production and *T*_a_ in an individual homeotherm. Within the thermoneutral zone (TNZ), the animal’s rate of heat production (metabolic rate), termed its BMR or RMR, is low and independent of *T*_a_. The lower and upper limits of the TNZ are the lower (*T*_LC_) and upper (*T*_UC_) critical temperatures. At *T*_a_ < *T*_LC_, the animal uses shivering and nonshivering thermogenesis (NST) to increase its rate of heat production as the environment becomes colder. Moreover, according to the standard model, at *T*_a_ < *T*_LC_, body insulation *I* is approximately constant, and accordingly the rate of metabolic heat production required for thermoregulation increases linearly as *T*_a_ falls. The absolute value of the slope of this increase is conductance *C* = 1/*I*. According to this model, extrapolation of the line segment below *T*_LC_ intersects the *x*-axis at a *T*_a_ equal to the animal’s deep body temperature, a consideration discussed later in this paper. In a city, rather than shivering and NST, furnaces or other building-heating devices are employed to increase the rate of heat production as the environment becomes colder at *T*_a_ < *T*_LC_. At *T*_a_ > *T*_UC_, an animal’s rate of heat production increases for complex reasons (Hill et al. 2016), including that panting and other mechanisms of increasing evaporative cooling require an investment of metabolic energy. In a city at *T*_a_ > *T*_UC_, the rate of heat production increases because of the energy cost of air conditioning and cooling fans

Figure 2 depicts a standard model of the energy-*T*_a_ curve of an individual mammal (Scholander et al. 1950a; McNab 2002; Hill et al. 2013, 2016). There is a range of *T*_a_s in which the animal’s rate of heat production (its metabolic rate) is constant regardless of *T*_a_. This range, the *thermoneutral zone* (*TNZ*), is bounded at its lower limit by the *lower critical temperature* (*T*_LC_) and at its upper limit by the *upper critical temperature* (*T*_UC_). The rate of heat production in the TNZ is the *basal metabolic rate*, which we call the *resting metabolic rate* (*RMR*) in application to cities. Within the TNZ, an animal varies its value of thermal insulation, *I*, as *T*_a_ varies. However, according to the standard model, an animal’s *I* is maximal and constant at *T*_a_s below the *T*_LC._ Because of this constancy of *I* below the *T*_LC_, the animal’s rate of heat production increases linearly as *T*_a_ decreases below the *T*_LC_. The absolute value of the slope of the energy-*T*_a_ curve at *T*_a_s below the *T*_LC_ is equal to *conductance*, *C*, which is the inverse of *I* (*I* = 1/*C*) (Scholander et al. 1950a; McNab 2002; Hill et al. 2013, 2016). Specifically, according to the standard model, the inverse of the absolute value of the slope is equal to the animal’s maximal value of thermal insulation, *I*. The insulation of a city is also expected to be constant and maximal at *T*_a_ < *T*_LC_ because it depends on the relatively unchanging physical structures of buildings that, at such *T*_a_s, are configured to minimize heat loss. Thus, the model developed for animal homeotherms (Fig. 2) can in principle be applied to cities.

Thermal insulation is defined in a standardized way for multiple applications. For any object that is warm on the inside and surrounded on the outside by cooler air, two factors determine the rate of heat loss. One is the magnitude of the temperature difference, *T*_inside_ − *T*_outside_. The other is the resistance to heat transfer posed by the physical structures that lie between inside and outside. Insulation (*I*), which formally refers to this resistance, is defined operationally by the following equation, analogous to Ohm’s law:

1 $$ I = \frac{{T_{inside} - T_{outside} }}{H} $$

where the numerator and denominator are measured simultaneously in steady-state and *H* represents the rate of internal heat production required to replace heat lost. In an animal, *H* is the animal’s metabolic rate (Scholander et al. 1950a; McNab 2002; Hill et al. 2013, 2016). In a building, *H* is the rate of heat production by the furnace or other devices that add heat to the building interior. Equation (1)—besides being used to understand the energy-*T*_a_ curves of animals and cities—is incorporated into all common measures of insulation. The equation is used to measure the insulative effectiveness of mammal furs (Scholander et al 1950b), the clo values that express clothing insulation (Gagge et al. 1941; Burton and Edholm 1955; Yan and Oliver 1996; Hill et al. 2013), and the *R* values that express the insulative effectiveness of construction materials (ASTM International 2017).

A critically important attribute of *I* is that it is a *holistic* measure of resistance to heat transfer. In a mammal, *I* depends on all the “resistive” factors that affect how readily the body loses heat in a cold environment. These factors include not only the animal’s pelage, but also its body posture and the vigorousness of blood flow to its body surface. When *I* is measured using Eq. (1), the effects of all these factors are taken into account and reflected in the value of *I* obtained. This holistic attribute is especially important for the study of city insulation. In a city, the city-wide *I* depends on a great many “resistive” factors: the types of materials built into the walls and attics of hundreds of variously shaped houses constructed by different people at different times, the types of window designs used in these varied houses, the spacing of living units (e.g., the proportion built with shared walls), the quality of structural maintenance over time, and other structural features. When *I* is measured using Eq. (1), all of these factors are taken into account and reflected in the value of *I* obtained, meaning that *I* provides a measure of the overall resistance to heat loss from a city’s buildings.

In this paper, we use the methods developed by Hill et al. (2013) to obtain energy-*T*_a_ plots for 42 North American cities that vary widely in thermal climate. To quantify thermal climate we use the average *T*_a_ during all days of the year for a 10-year period, *T*_10-year_, a metric that ranges from − 10.8 to + 25.5 °C in the 42 cities studied. We ask the question: does city-wide *I* vary significantly with *T*_10-year_? Our a priori null hypothesis is that there is no significant relationship between *I* and *T*_10-year_. Our alternative hypothesis is that *I* increases as *T*_10-year_ decreases. This alternative relationship is what would be expected if people constructing cities in cold climates have incorporated more features that impede heat loss than people constructing cities in thermally moderate climates.

## Materials and methods

We approached the electric and natural-gas utilities of over 150 mid-sized cities in North America (population: 2000–155,000) to request daily, city-wide usage data for an entire year, and here we report on all 42 cities in which the primary energy sources for home heating are electricity and natural gas, and for which the utilities agreed to provide data. In the process of gathering these data, we sought cities with widely different exposure to low *T*_a_, as judged by published data on annual average air temperature, measured specifically as *T*_10-year_ (we calculated *T*_10-year_ for all cities using a standardized method described later). We recognized six categories of published annual average temperature: < − 4 °C, − 4 °C to + 2 °C, 2–8 °C, 8–14 °C, 14–20 °C, and 20–26 °C. Then, as we sought cities for inclusion in this study, we maintained two objectives: (1) obtain data on as many cities as possible, but also (2) include, to the extent possible, approximately equal numbers of cities in the six temperature categories. We limited our search to mid-sized cities because large cities often have indefinite physical and functional boundaries, and because the inclusion of large cities would have substantially increased among-city heterogeneity in the urban-heat-island effect (Oke 1973, 1982).

Prior to approaching the utilities in a city, we used publicly available satellite images and narratives (e.g., city descriptions at city websites) to assess whether large, energy-intensive factories—or other industrial or military facilities—were present in the city. We did not approach cities in which we were able to identify such facilities. We also focused on city boundaries and did not pursue cities that we became aware were seamlessly connected to adjacent cities. If we approached the utilities in a city, we queried utility personnel about the possible presence of heavy industrial or military customers and withdrew our request if we learned of their presence.

Approached candidate cities were often unusable because one of the utilities could not or would not provide the data needed. If the utilities expressed willingness to cooperate but could not provide the exact types of energy-use data we sought, we sometimes modified our request. Most notably, in two far-northern cities (Inuvik, Utqiagvik) we obtained data on energy use at weekly or monthly resolution because data at daily resolution were unavailable.

Our target year for data was 2013, and for 35 cities we report data for that year. We also include (and reanalyze) the six cities we earlier studied (Hill et al. 2013), for which data apply to 2009 or 2010, and one city with data for 2014.

For each city, we calculated city-wide, within-building heat production during each 24-h calendar day—symbolized *H*_city_—as the sum of the heat equivalents of electric and natural-gas energy used that day (or, analogously, if the resolution was weekly or monthly, we summed heat production over all 24-h days in a week or month). Some cities (particularly in relatively warm regions) do not use natural gas, and in those cases we calculated daily heat production from electric data alone. To produce an energy-*T*_a_ plot, daily *T*_a_ must be known for each calendar day. To obtain data on the average *T*_a_ during each day, we used MapServer at the Utah Climate Center (Utah State University) to access temperature records for cities in the United States. For cities in Canada, we used either the Government of Canada Historical Climate Data database or the Environment Canada database. For a typical city, using these methods, we obtained 365 *x*, *y* data points (one per calendar day in a year), where *x* is daily average *T*_a_ and *y* is daily city heat production, *H*_city_. In Utqiagvik (formerly Barrow), AK, we obtained 49 weekly data points. In Inuvik, NWT, we obtained 36 monthly data points by combining data for three adjacent years (2013–2015).

All statistical methods were planned in detail a priori. After data were obtained, the data were processed strictly according to the a priori plan.

To calculate city-wide *I*, we use two methods, which differ in how effectively they address two types of limitations in the data available (see “Discussion”). We term these the “point-by-point” and “slope” methods.

### Point-by-point method to calculate city-wide *I*

The point-by-point method is a straightforward application of Eq. (1), which—in the study of cities—takes the form: *I* = (*T*_LS_ − *T*_a_)/*H*_city_, where *T*_LS_ is the air temperature in the living spaces of buildings. As already described, for each city we have daily data on *T*_a_ and *H*_city_ for an entire year. Accordingly, we can calculate city-wide *I* for each day (thus, “point-by-point” method), limiting ourselves to days when *T*_a_ ≤ *T*_LC_ because, as described earlier, *I* is expected to be constant at such *T*_a_s, meaning all such estimates of *I* are estimating the same value. The obvious problem with the application of this method is the lack of empirical knowledge of the temperature maintained in living spaces, *T*_LS_. However, we do know that people in North America tend to set thermostats near 20 °C in the cold seasons of the year (Huchuk et al. 2018). We carry out the calculations three times with three *T*_LS_ estimates, looking for convergences.

### Slope method

The slope method explicitly addresses a different limitation: the problem that city population size, *P*, affects *I*, but there is no established theory for taking *P* into account. The slope method addresses this problem by calculating a derivative, unitless measure of insulation that is independent of *P*. To apply this method to a city, we calculate *C*, the absolute value of the slope of the linear regression of *H*_city_ versus *T*_a_ below *T*_LC_ (see Fig. 2). *C* itself provides a measure of city-wide *I* because *I* = 1/*C*. With the regression, however, we can calculate the regression-based ratio of energy use at two different *T*_a_s, a unitless measure of insulation independent of city size. We use the regression to calculate the ratio: estimated *H*_city_ at *T*_a_ = − 20 °C/estimated *H*_city_ at *T*_a_ = 10 °C, symbolized *H*_city(-20)_ /*H*_city(10)_ (see “Discussion” for explanation of *T*_a_s chosen). In addition to being unitless, this ratio provides a measure that is easily understood by nonspecialists.

All statistical calculations were carried out in IBM SPSS Statistics (version 26) except that we used the Segmented package (for segmented regression; Muggeo 2008) in R (R Project for Statistical Computing; R Core Team 2021) to estimate the lower critical temperature, *T*_LC_, of each city (SPSS lacks a procedure for segmented regression). To keep the calculation of *T*_LC_ as straightforward as possible and minimize the chances of confounding factors (see “Discussion”), we wanted the data analyzed by Segmented to include only one breakpoint. Thus we used the data for *T*_a_ ≤ 19 °C to calculate *T*_LC_ and specified one breakpoint in the application of Segmented. We ran Segmented three times for each city, seeding the iterative calculations with three starting approximations of *T*_LC_, 10 °C, 12 °C, and 14 °C. Where the estimate of *T*_LC_ differed because of starting value, we used minimization of the Akaike information criterion (AIC) to determine which estimate of *T*_LC_ to use.

To calculate the regression of the energy-*T*_a_ relationship below thermoneutrality for a city, we first calculated an upper *T*_a_ limit for the data to be used. Specifically, to minimize chances of including data points from the TNZ (see Fig. 2), we calculated the upper *T*_a_ limit for the regression as our estimated *T*_LC_ minus 1 SE of the *T*_LC_ estimate. We carried out linear least-squares regression on the data for all days in which *T*_a_ was less than or equal to this upper limit.

Although the energy-*T*_a_ relationship at high *T*_a_s is not of central concern for this paper, we estimated the upper critical temperature of each city, *T*_UC_, and the slope above *T*_UC_ in ways analogous to the procedures just described. To estimate *T*_UC_ we used the data for *T*_a_s ≥ 14 °C and specified one breakpoint in Segmented. We ran Segmented three times for each city (seeding with starting approximations of 18 °C, 20 °C, and 22 °C) and resolved ambiguities using AIC. To estimate the regression of the energy-*T*_a_ relationship above thermoneutrality, we calculated a lower *T*_a_ limit for the data to be used as *T*_UC_ plus 1 SE. We then carried out linear least-squares regression, using the data for all days when *T*_a_ was equal to or higher than this lower limit.

To calculate RMR, the mean daily energy consumption in the TNZ of each city, we averaged the data for all days when the *T*_a_ was (1) more than 1 SE above *T*_LC_ (using the SE of the estimate for *T*_LC_) and (2) more than 1 SE below *T*_UC_ (using the SE of the estimate for *T*_UC_). There were many cities for which Segmented detected no *T*_UC_ (i.e., no breakpoint representing *T*_UC_). In such cases, we calculated the mean daily energy consumption in the TNZ using the data for all days meeting criterion (1).

Throughout the rest of this paper, we use a short-hand terminology for referring to data boundaries. Where we speak of data gathered on days when *T*_a_ was “below the *T*_LC_,” in fact the days were those with *T*_a_ below *T*_LC_ minus 1 SE of the *T*_LC_. Similarly, when we speak of data “above the *T*_UC_,” the data were actually above the *T*_UC_ plus 1 SE of the *T*_UC_.

For each city, we obtained three additional types of information used in our analysis: population size, number of electric accounts, and *T*_10-year_. For cities in the United States, we obtained population size from the US Census Bureau (2010 census); for cities in Canada, we used Statistics Canada. To obtain the number of electric accounts, we asked the electric utility.

To calculate *T*_10-year_ we used data acquired through MapServer (Utah Climate Center), the Government of Canada Historical Climate Data database, or the Environment Canada database. Barring lapses in data records, our target 10-year period was typically 2004–2013, and we averaged the *T*_a_ data for all days in that period. If data were missing for some of those years, to obtain 10 years of data, we included data (if available) from the adjacent years for which data were available and that were closest in time to the target 10-year period. For cities in which long-term *T*_a_ records have not been maintained, we used data for the closest cities, of similar size and altitude, where data are available. For three cites, we could not obtain 10 years of data near our target years and used 8 years (Fort Macleod) or 5 years (Cairo; Thomasville); for Inuvik, the only suitable data were “climate normal” data for 1981–2010.

## Results

**Fig. 3:**
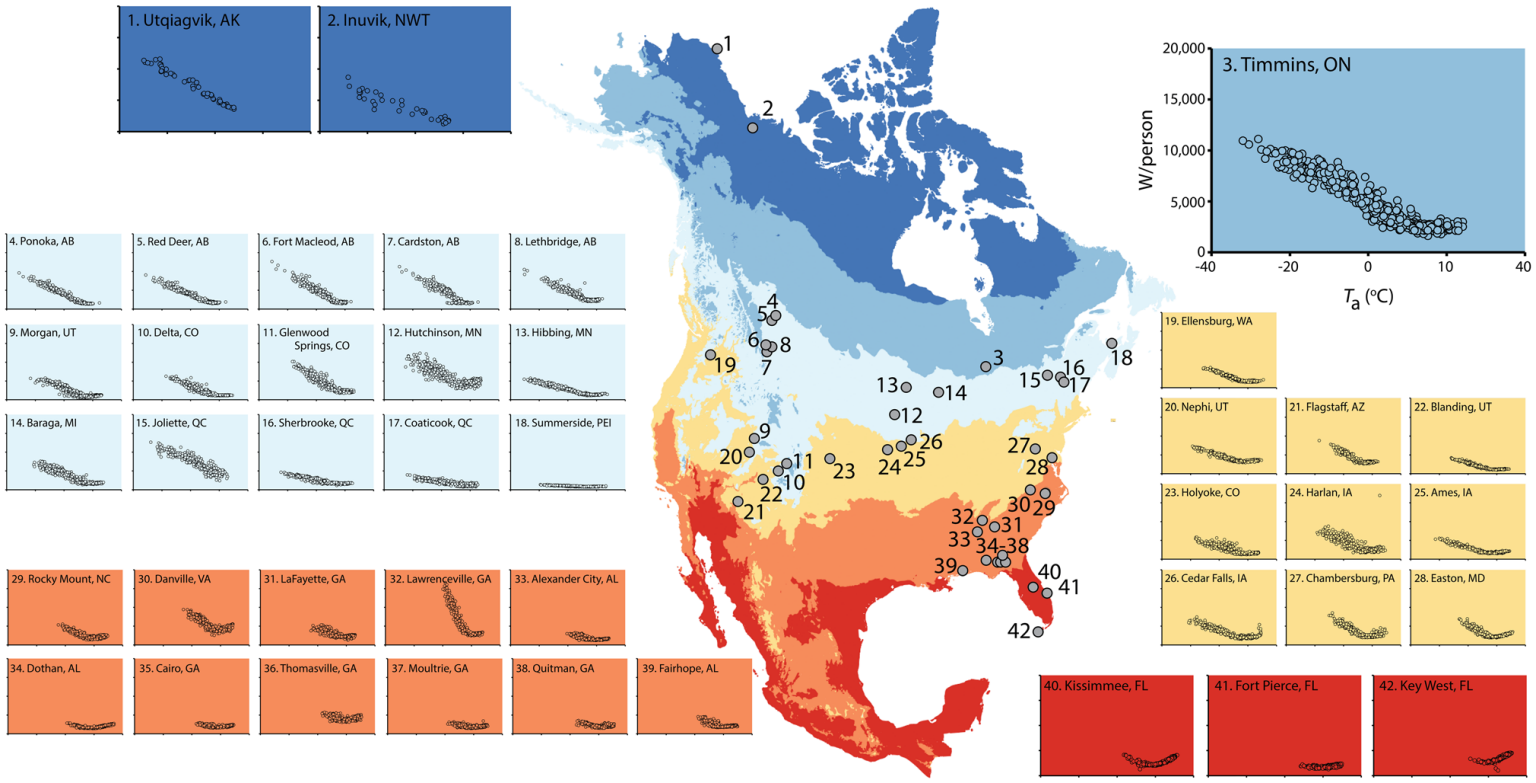
Energy-*T*_a_ plots for 42 North American cities. Each plot depicts the city rate of heat production *H*_city_ normalized to population size *P* (i.e. W/ person) as a function of average daily outside air temperature (*T*_a_) for an entire year. For most cities, the plot covers ca. 365 days, with each symbol representing one day. Axes of all plots are scaled identically to the scaling shown in the enlarged plot for Timmins. To display the geographical distribution of the cities (important information addressed in the “Discussion”) and to help identify cities that are found in similar thermal climates, the locations of cities are plotted on a map color-coded to show average annual air temperature based on historical climate records (Wang et al. 2016): dark blue, < − 4 °C; medium blue, − 4 to + 2 °C; light blue, 2–8 °C; yellow, 8–14 °C; orange, 14–20 °C; and red, 20–26 °C. These are the same six categories of annual average air temperature employed in our data acquisition protocol as described in “Materials and methods”. Annual average air temperature depends on altitude and proximity to seacoasts as well as latitude

The energy-*T*_a_ curves for all 42 cities are presented in Fig. 3, where the cities are plotted at their geographic locations on a map that delineates zones of average annual air temperature based on historical climate records. Table 1 presents additional information on each city: altitude, *T*_10-year_, and population *P*. Although the zones of average annual air temperature in Fig. 3 provide useful background information, for the statistical analysis of the data each city’s specific *T*_10-year_ (Table 1), not zone, was used.

**Table 1:** Altitude, *T*_10-year_, and population size of the cities

City	Altitude (m)	*T*_10-year_ (°C)	Population (*P*)
Alexander City, AL	200	17	14,900
Ames, IA	280	9.3	59,000
Baraga, MI	200	3.8	2100
Blanding, UT	1840	11.9	3400
Cairo, GA	60	19.5	9600
Cardston, Alberta	1190	5.7	3300
Cedar Falls, IA	290	8.9	39,300
Chambersburg, PA	200	11.7	20,300
Coaticook, Quebec	260	6.1	9300
Danville,VA	170	14.9	43,100
Delta, CO	1530	10.8	8900
Dothan, AL	100	19.6	65,500
Easton, MD	10	15.3	15,900
Ellensburg, WA	540	10.4	18,200
Fairhope, AL	40	19.9	15,300
Flagstaff, AZ	2100	8.1	65,900
Fort Macleod, Alberta	950	5.2	3100
Fort Pierce, FL	0	22.9	41,600
Glenwood Springs, CO	1810	8.7	9600
Harlan, IA	380	9.5	5100
Hibbing, MN	410	3.6	16,400
Holyoke, CO	1140	10	2300
Hutchinson, MN	330	6.9	14,200
Inuvik, NWT	70	− 8.2	3500
Joliette, Quebec	60	6.2	19,600
Key West, FL	0	25.5	24,600
Kissimmee, FL	20	22.5	59,700
LaFayette, GA	250	14.9	7100
Lawrenceville, GA	260	16.6	28,600
Lethbridge, Alberta	930	6.4	83,500
Morgan, UT	1550	7.8	3700
Moultrie, GA	100	19.5	14,300
Nephi, UT	1570	10.4	5400
Ponoka, Alberta	860	3	6800
Quitman, GA	60	20.3	3900
Red Deer, Alberta	850	2.9	90,600
Rocky Mount, NC	30	16	57,500
Sherbrooke, Quebec	250	4.9	154,600
Summerside, PEI	10	6.2	14,800
Thomasville, GA	60	19.5	18,400
Timmins, Ontario	290	2.3	43,200
Utqiagvik, AK	4	− 10.8	4212

*T*_10-year_ is the average *T*_a_ recorded over a 10-year period (see “Materials and methods”)

We analyzed the potential correlation between *T*_10-year_ and *P* for the 42 cities. The Pearson correlation coefficient between the two variables is 0.04 (*p* > 0.8), indicating no relationship. A scatterplot of the two variables likewise suggests that *T*_10-year_ and *P* are completely unrelated in our dataset.

### Point-by-point method

For each city, we calculated *I* = (*T*_LS_ − *T*_a_)/*H*_city_ for each day when *T*_a_ was below *T*_LC_, assuming in three sequential analyses that *T*_LS_ was 20 °C (Huchuk et al. 2018), 17 °C, or 23 °C (see “Materials and methods”, and “Discussion”, for explanation). Table 2 lists for each city the *T*_LC_ and the number of days, *N*, when *T*_a_ was below *T*_LC_; *N* is the number of estimations of *I* provided by the point-by-point method at each *T*_LS_.

**Table 2:** Results of the two methods

City	*T*_LC_ (°C)	*N* below TNZ	Result of point-by-point method: mean *P*-normalized *I* (°C∙person/MW) with *T*_LS_ = 20 °C	Result of slope method: *H*_city(-20)_/*H*_city(10)_
Alexander City, AL	14.5	119	5500	2.24
Ames, IA	11.9	180	6520	2.73
Baraga, MI	14.5	243	4910	2.82
Blanding, UT	14.8	221	6530	2.98
Cairo, GA	11.6	37	5420	2.11
Cardston, Alberta	14.0	285	4090	3.49
Cedar Falls, IA	13.6	205	5250	2.61
Chambersburg, PA	12.2	175	3600	3.15
Coaticook, Quebec	16.2	251	8830	2.06
Danville,VA	14.5	162	2090	2.48
Delta, CO	9.2	144	5540	3.36
Dothan, AL	11.8	64	5700	2.51
Easton, MD	13.1	165	3590	3.17
Ellensburg, WA	15.0	231	4520	2.78
Fairhope, AL	12.2	67	3730	2.85
Flagstaff, AZ	10.9	218	3420	3.12
Fort Macleod, Alberta	13.7	251	4000	3.52
Fort Pierce, FL	12.3	10	5080	2.29
Glenwood Springs, CO	13.7	218	3470	3.12
Harlan, IA	15.6	221	4120	2.52
Hibbing, MN	13.5	249	7510	2.76
Holyoke, CO	8.0	159	5950	2.01
Hutchinson, MN	13.6	222	3180	2.15
Inuvik, NWT	–	36	6090	2.89
Joliette, Quebec	14.8	233	2580	1.90
Key West, FL	17.9	16	1450	1.85
Kissimmee, FL	11.9	19	3770	3.35
LaFayette, GA	17.2	193	3740	2.29
Lawrenceville, GA	14.0	137	1330	4.39
Lethbridge, Alberta	14.3	222	3960	2.69
Morgan, UT	14.8	245	7430	4.21
Moultrie, GA	15.0	75	4830	2.00
Nephi, UT	11.1	180	3930	2.08
Ponoka, Alberta	13.1	257	5100	3.60
Quitman, GA	19.4	126	2550	2.10
Red Deer, Alberta	12.0	237	5270	3.19
Rocky Mount, NC	15.0	145	3890	2.75
Sherbrooke, Quebec	10.1	216	8240	2.32
Summerside, PEI	11.3	211	20,000	1.58
Thomasville, GA	19.0	143	1650	1.75
Timmins, Ontario	10.2	243	4230	3.28
Utqiagvik, AK	–	49	3930	3.09

Raw data for Inuvik and Utqiagvik were monthly and weekly, respectively

**Fig. 4:**
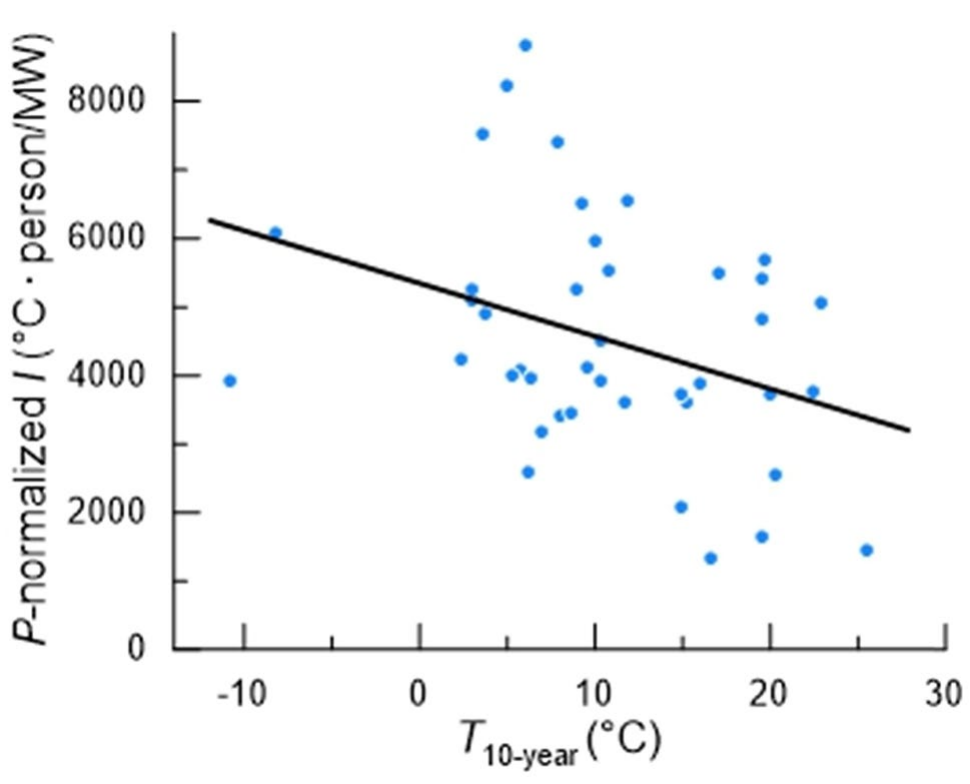
Results of the point-by-point method: average *P*-normalized *I* plotted versus *T*_10-year_ for 41 cities. Each symbol represents one city. For each city, *P*-normalized *I* = (*T*_LS_ − *T*_a_) ·*P*/*H*_city_ was calculated for each day when *T*_a_ was below *T*_LC_, assuming *T*_LS_ = 20 °C, and all days were averaged. *T*_LC_ and the number of days analyzed are tabulated in Table 2. The coefficients of the regression are in Table 3

To analyze the relation between the daily measures of city-wide *I* and *T*_10-year_, our initial plan had been to remove effects of city population size, *P*, by analysis of covariance. However, scatter-plots revealed that assumptions of analysis of covariance (e.g., normality, homoscedasticity, and linearity) were not always met. Thus, following our a priori strategy, we calculated the average *P*-normalized *I* [see Eq. (1)] for each city by dividing (*T*_LS_ − *T*_a_) by *H*_city_/*P* for each day and then averaging the data over all *N* days at each assumed *T*_LS_. Given that thermostats are commonly set near 20 °C during the cold seasons of the year in North America (Huchuk et al. 2018), we first analyzed the data with *T*_LS_ = 20 °C (Table 2). The datum for one city (Summerside, PEI) was a statistical outlier and thus not included in further analysis. After removing it, we fitted a linear regression (Fig. 4), which indicates a statistically significant inverse relationship between city-wide *I* and *T*_10-year_, although the regression explains just 9.6% of variance in *I* (Table 3). We repeated the analysis assuming *T*_LS_ = 17 °C and *T*_LS_ = 23 °C. Assumed *T*_LS_ affects the statistical relationship between *I* and *T*_10-year_ (Table 3). As *T*_LS_ is set to higher values, the absolute value of the estimated slope decreases, the fraction of variance explained by the regression decreases, and certainty in a non-zero slope decreases (Table 3).

**Table 3:** Results of the point-by-point method

Assumed living-space temperature, *T*_LS_ (°C)	Slope (person/MW)	Intercept (°C∙person/MW)	*p*: probability that slope is 0	Adjusted *r*^2^
17	− 91.2	4615	0.0012	0.19
20	− 76.1	5364	0.014	0.096
23	− 61.0	6113	0.062	0.036

Regression results for linear regressions carried out on the relationship between average *P*-normalized, city-wide *I* and *T*_10-year_ for 41 cities under three assumed values of *T*_LS_. *p* is the one-tailed probability because our a priori alternative hypothesis was directional

### Slope method

To implement this method, we first calculated the linear regression for the total rate of heat production (*H*_city_) as a function of *T*_a_, at *T*_a_ < *T*_LC_, for each of the 42 cities (see Supplementary Table 1 for slope, intercept, and *r*^2^). In all cases, the regression had a statistically significant slope different from 0 (*p* < 0.0025, except *p* = 0.01 for Fort Pierce).

**Fig. 5:**
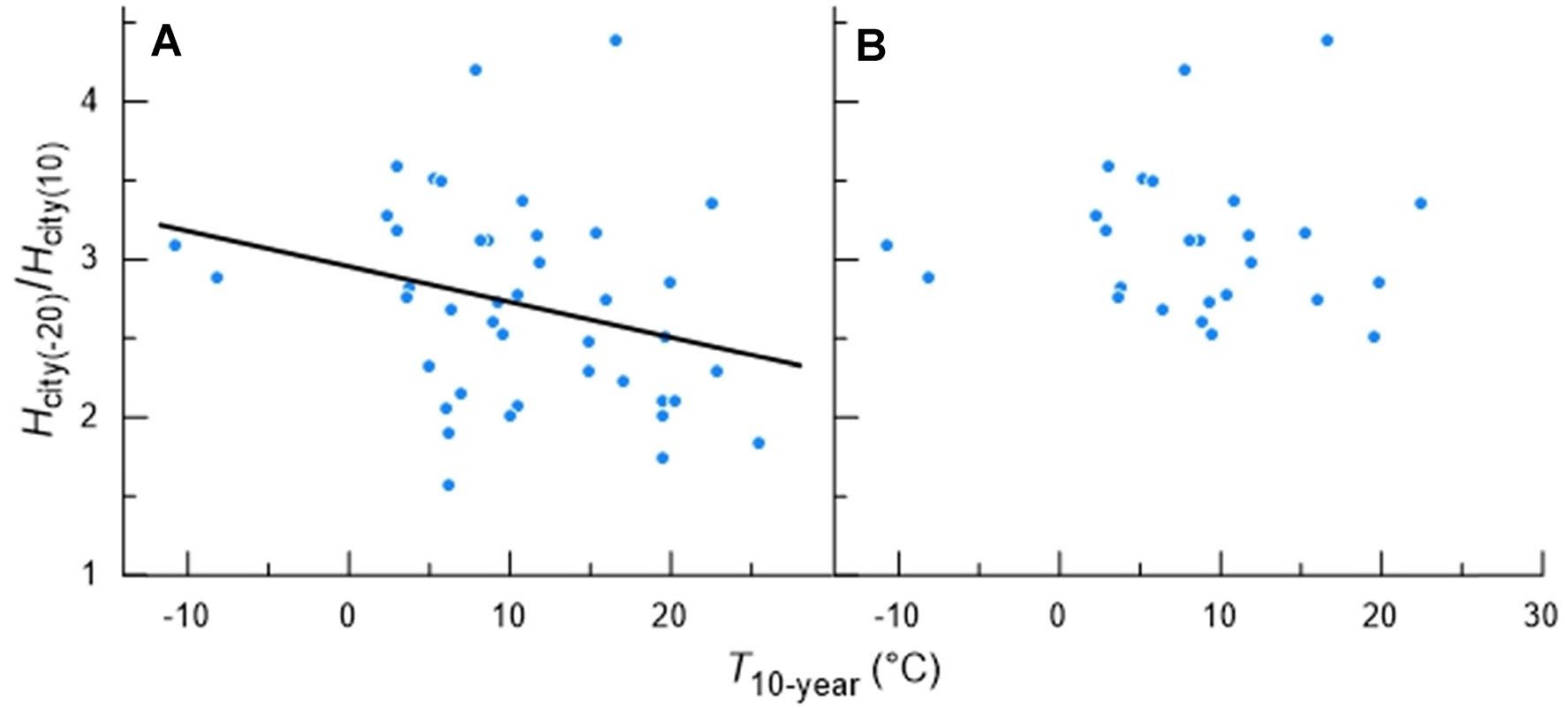
Results of the slope method: *H*_city (-20)_ /*H*_city (10)_ plotted in relation to *T*_10-year_. Each symbol represents one city. For each city, the linear regression of *H*_city_ as a function of *T*_a_, for all days when *T*_a_ < *T*_LC_, was calculated (see Supplementary Table 1). Using the regression, predicted *H*_city_ at *T*_a_ = − 20 °C divided by predicted *H*_city_ at *T*_a_ = 10 °C was calculated as a measure of city insulation. A includes 42 cities. The slope of the regression is significantly different from zero: *H*_city (-20)_ /*H*_city (10)_ = 2.95−0.022·*T*_10-year_ (*p* = 0.044). B includes the subset of 26 cities for which the *x*-intercept of the city regression is < 30 °C

Using the *H*_city_-versus-*T*_a_ regression for each city, we calculated the ratio of predicted *H*_city_ at *T*_a_ = − 20 °C divided by predicted *H*_city_ at *T*_a_ = 10 °C, symbolized *H*_city(-20)_/*H*_city(10)_, to obtain a unitless index of city insulation (Table 2). Figure 5A shows this index as a function of *T*_10-year_ for all 42 cities. The slope of the regression (− 0.022 per °C) is significantly different from 0 (*p* = 0.044, one-tailed), although the adjusted r^2^ is 0.048, indicating that the regression explains just 5% of variance. According to the standard model (Fig. 2), the slope of the *H*_city_-versus-*T*_a_ regression of a city at *T*_a_ < *T*_LC_ is approximately equal to 1/*I* if the regression extrapolates to intersect the *x*-axis at a temperature approximating *T*_LS_ (Scholander et al. 1950a; McNab 2002; Hill et al. 2013, 2016). The *x*-intercept for some cities is too high to plausibly approximate *T*_LS_. Following our a priori plan, we thus winnowed the set of cities analyzed to include only those 26 cities for which *x*-intercept < 30 °C, presented in Fig. 5B. Within this subset of cities, there is no statistically significant relationship between *H*_city(-20)_ /*H*_city(10)_ and *T*_10-year_ (*p* = 0.95).

Related to the slope method is the concept that the predicted value of *H*_city_ at a specific low *T*_a_ could also serve as an index (inverse) of city resistance to heat loss. Thus, for each of the 42 cities in our full dataset, we calculated *H*_city_ at *T*_a_ = − 20 °C using the *H*_city_-versus-*T*_a_ regression (Supplementary Table 1). Recognizing that city population size, *P*, helps determine *H*_city_, we carried out an analysis of covariance to examine the potential relationship between *H*_city_ at *T*_a_ = − 20 °C and *T*_10-year_. *P* explains a highly significant part of the total variance (*p* < 0.005). However, there is no evidence of a significant relationship between *H*_city_ at *T*_a_ = − 20 °C and *T*_10-year_ (*p* = 0.18).

### Metrics in the TNZ and at *T*_a_ > *T*_UC_

The Segmented routine in R identified a breakpoint corresponding to *T*_UC_ in the energy-*T*_a_ curves for 25 cities, but not the other 17 cities. Supplementary Table 2 summarizes the estimates of *T*_UC_, estimates of RMR/person (mean *H*_city_/*P* in the TNZ), and—for cities with a defined *T*_UC_—the regression coefficients for regression between *H*_city_ and *T*_a_ at *T*_a_ > *T*_UC_.

For statistical analysis of RMR, the regression of mean total city-specific RMR (mean *H*_city_ in the TNZ) as a function of *T*_10-year_ was examined with *P* as a covariate. Although the effect of *P* was significant (*p* < 0.001), that of *T*_10-year_ was not (*p* = 0.61).

## Discussion

**Fig. 6:**
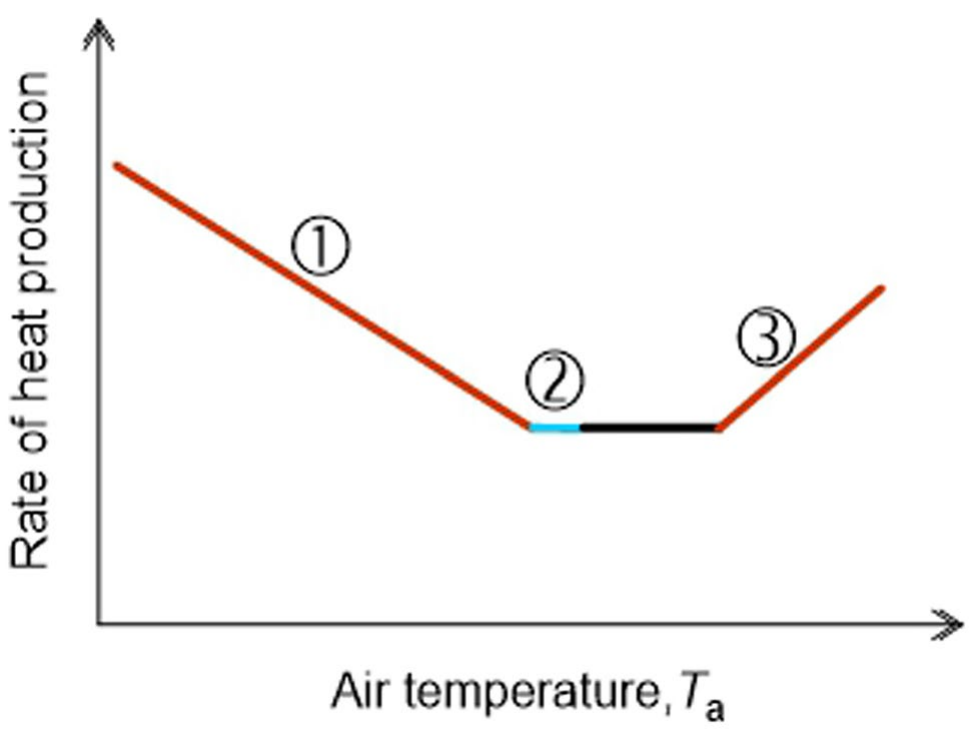
Line segments in the energy-*T*_a_ relationship of a city. Shown is a generic energy-*T*_a_ curve on which three parts are identified and numbered so that they can be referenced specifically in the accompanying discussion: ①, the part of the curve below the thermoneutral zone (TNZ); ②, the initial (i.e., low *T*_a_) part of the TNZ; and ③, the part of the curve above the TNZ. Compare with Fig. 2

The summary of data in Fig. 3 abundantly confirms that, as first demonstrated by Hill et al. (2013) (see also Meehan 2012), individual North American cities typically exhibit energy-*T*_a_ curves similar to those of animal homeotherms. Cities do this because the basic principles of heat exchange that apply to homeotherms also apply to cities. General trends correlated with climate zones are readily apparent (Fig. 3). Virtually all cities manifest segment ① of the full energy-*T*_a_ curve (see Fig. 6) because this part reflects the increasing energy cost of maintaining a steady internal temperature (*T*_LS_) by use of heating systems as *T*_a_ decreases below *T*_LC_. However, southern Florida is so warm that Ft. Pierce and Key West barely express segment ①. Conversely, cities in the Far North such as Inuvik and Utqiagvik (previously Barrow) experience such cold environments that they do not express segment ②, the part of the TNZ at *T*_a_s immediately above the *T*_LC_; that is, these cities do not express the TNZ. Cities in high mountains (see Table 1 for altitude data) tend to express a narrower part of the TNZ than lowland cities at similar latitudes. Segment ③ of the energy-*T*_a_ curve (see Fig. 6) reflects the use of air conditioning and its increasing energy cost as *T*_a_ increases above *T*_UC_. Cities in the two warmest climate zones (orange and red, Fig. 3) vary in how vividly they express this part of the curve, some cities expressing it vividly (e.g., Key West), whereas others barely express it. Probably, a significant factor in this variation is the percentage of residences in a city that employ air conditioning; an entire city will strongly express segment ③ only if a substantial subset of residences employ air conditioning. In the set of 42 cities, a few exhibit atypical curves (e.g., Lawrenceville, Summerside). In this study, we did not reject cities because of their energy-*T*_a_ curves. We applied rigorous quality standards in sourcing data and calculating results from data, but if a city met these quality standards, it has been included in our analysis. As stressed earlier, we defined every step of our data analysis a priori; thus, the nature of results was never a factor in the results reported.

The paucity of northern cities in our dataset is evident in Fig. 3. This paucity reflects a fundamental limitation for studies using our methods. Namely, energy data at defined temporal resolution are not equally available from the relatively cold and relatively warm parts of North America. To a dramatic degree, as we turned to colder and colder parts of the continent in our search for city data, we found the data to be more and more difficult to obtain. The reason is that in many cold-climate regions, households rely principally on fuel oil or other heating fuels delivered in bulk. In such cases, the times of use of fuel to produce heat are not sufficiently defined to be correlated with *T*_a_ records.

### Relation between city-wide insulation below the TNZ and thermal climate

Considered in its entirety, this research indicates that in North American cities built in a wide range of thermal climates (*T*_10-year_ between − 11 °C and + 26 °C), city-wide *I* tends to be higher, the colder the climate.

To analyze the data with the point-by-point method, we used three estimated values of *T*_LS_ (living-space temperature). An underlying assumption is that *T*_LS_ is independent of *T*_a_ at *T*_a_ < *T*_LC_, an assumption supported by the data for the heating seasons in Huchuk et al. (2018). The regression between population-normalized city-wide *I* and *T*_10-year_ is statistically significant for *T*_LS_ = 17 °C (*p* = 0.0012) and *T*_LS_ = 20 °C (*p* = 0.014), although moderately short of statistical significance at *T*_LS_ = 23 °C (*p* = 0.062) (Table 3). Certainly, 17 °C and 20 °C would seem to be more plausible values than 23 °C for average thermostat settings in cold seasons of the year. The results of our analysis with the unitless slope method also tend to point to a significant inverse relationship between city-wide *I* and *T*_10-year._ In that analysis, we quantified *I* as the ratio of two regression-calculated values of *H*_city_: *H*_city(-20)_ /*H*_city(10)_. The regression for all 42 cities (Fig. 5A) is statistically significant (*p* = 0.022). Although analysis with just cities that adhere to the *x*-intercept expectation of the standard *H*_city_-*T*_a_ model does not yield a significant regression (Fig. 5B), the fact remains that—quite apart from any model—the index *H*_city(-20)_ /*H*_city(10)_ is an instructive, accessible metric in its own right, reflecting the extent to which a city’s energy use must be elevated for thermoregulation at *T*_a_ = − 20 °C relative to *T*_a_ = 10 °C. Thus the full set of data on *H*_city(-20)_ /*H*_city(10)_ is pertinent (Fig. 5A).

Considering all the data available and the results of both the point-by-point and slope methods, we conclude that as people have constructed cities in various climates in North America, they have—at a community level—tended to build better-insulated living spaces in cold climates than moderate ones: a trend that aids energy conservation. Variance is high, however, and statistically speaking, *T*_10-year_ explains only a small proportion of total variance in city-wide *I*: a mean of 11% in the point-by-point method and 5% in the slope method. Thus, the evidence for adaptive modulation of city-scale thermal insulation might be aptly described as suggestive but tentative. The high variance might indicate that cities in North America have varied a great deal in the extent to which insulation has been a priority in city development. The high variance certainly presents an obstacle to the accurate elucidation of any trends that exist.

Focusing on our estimates of city-wide *I* in the point-by-point analysis (estimates expressed in units of measure, rather than being unitless), we can address a significant question: Is the slope of the regression between *I* and *T*_10-year_ (Fig. 4) great enough that the increasing *I* at low *T*_10-year_ entirely offsets the effect of the increasing cold? Here we contribute just a relatively simple analysis based on the definition of *I* [Eq. (1)], assuming *T*_LS_ to be 20 °C (Huchuk et al. 2018). As a starting point, if we exclude extremes—notably Key West and the two extreme-cold cities (Inuvik and Utqiagvik)—we can calculate from published databases (for the U.S., *U.S. Climate Data*; for Canada, *Environment Canada Climate Normals 1981–2010*) that the average daily low *T*_a_ in December-February at our five warmest cities (average *T*_10-year_ = 21.0 °C) is 7 °C, whereas at our five coldest cities (average *T*_10-year_ = 3.1 °C), the average daily low *T*_a_ in December-February is − 15 °C. Combining the energy-*T*_a_ plots of the five warmest cities, we calculate a combined city-wide *I*_warm cities_ of 4200 °C·person/MW and estimate the energy cost (W/person) at *T*_a_ = 7 °C for those cities taken as a group. We then ask what the value of *I* in the five coldest cities, *I*_cold cities_, would need to be for the energy cost in the cold cities (W/person) at *T*_a_ = − 15 °C to be the same; this is the *I* in the cold cities that would be required to completely offset the increased cold there so they would have no higher energy cost than the warm cities. By this analysis, *I*_cold cities_ would need to be 2.7 times higher than *I*_warm cities_ to achieve this end. In actuality, the regression we calculated from our city energy-*T*_a_ curves (Fig. 4) indicates that the average *P*-normalized *I* is 1.4 times higher in our cold cities (again excluding Inuvik and Barrow) than our warm cities (excluding Key West). The trend we detect is thus only about half as great as would be required for increased city-wide *I* to offset fully the effects of thermal climate.

Prior to this research, there were already reports in the literature that, taking a reductionistic approach, focused on particular variables in home construction that affect insulation. For example, Healy (2003) reported that 85–100% of houses in Finland, Norway, and Sweden have cavity insulation in the walls, whereas only 6–68% in other European countries do. Holden and Norland (2005) presented evidence that per-capita energy use in Norway is lower in living units with shared walls than in stand-alone living units, although the difference is small in modern (post-1980) construction.

However, for energy analysis on regional or continental spatial scales, it is important to know holistically the resistance to heat loss of the large assemblages of houses and other occupied buildings in entire cities. Moreover—and just as important—this resistance does not depend solely on individual reductionistic properties (e.g., insulating material in house walls). Resistance to heat loss at city scale depends on many factors, including the totality of materials used to build dwellings at various stages in a city’s history, dwelling geometry, and maintenance. As explained in the Introduction, city-wide *I*—as we use the concept in this report—is a holistic measure of resistance to heat loss that takes all these considerations into account. Thus, city-wide *I* is the operationally relevant property that energy analysts need to know for understanding how efficiently energy consumed for thermoregulation is used to create desired temperatures in living spaces. Our methods estimate this city-wide *I*.

### Statistical methods for estimating city-wide *I*

Our methodological approach has two great strengths. It (1) uses publicly available information to (2) quantify a hard-to-measure property, city-wide *I*, of considerable potential importance for understanding city energy use and the effectiveness of energy-conservation efforts. Our approach to measuring city-wide *I* evades intrusive house-by-house insulation measurements that would be complex, expensive, and politically tenuous. Because of its practicality and relatively low cost, our approach provides perhaps the only immediately available and realistic means of measuring city-wide *I* on regional or continental scales.

However, difficult challenges [e.g., taking account of the influence of *P* (Packard and Boardman 1988)] are encountered in extracting information on city-wide *I* from energy-*T*_a_ plots. Thus, this paper is as much an exploration into techniques for calculating city-wide *I* from energy-*T*_a_ plots as it is a study of the relationship between *I* and *T*_10-year_.

From the outset, we have put a high priority on rule-based data processing, using rules articulated a priori. We tested and refined analytical methods by working on a subset of 6 cities prior to carrying out any analysis on the other 36 cities. Then, when we analyzed the full dataset, we followed the well-defined protocols established by the preliminary work.

A critical first step in any analysis is the estimation of *T*_LC_. Only a subset of a city’s data are pertinent to calculating city-wide *I*, and that subset is defined by the calculated value of *T*_LC_ because *I* is expected to be constant only at *T*_a_ < *T*_LC_. We, in fact, calculated city-wide *I* using the subset of data for *T*_a_ < *T*_LC_ − 1 SE of *T*_LC_ (see “Materials and methods”). This approach was adopted to minimize the odds of unintentionally including data from the TNZ in the data subset used to calculate *I*.

In both the present report and Hill et al. (2013), we have used procedures for segmented regression to estimate *T*_LC_. These procedures define breakpoints in a city’s energy-*T*_a_ data. Hill et al. (2013) used SAS (version 9.2) to estimate *T*_LC_ and *T*_UC_ simultaneously, and the procedure behaved in a nuanced manner, rarely producing a result when the human eye suggested there was no breakpoint. Here we used the Segmented package in R, which behaved differently. Comparing the two procedures, both of which are iterative, Segmented is notably more deterministic; instructed to find one breakpoint, it nearly always finds one, even in cases where the human eye can barely perceive a breakpoint; instructed to find two breakpoints, it finds two. We used separate analyses to identify *T*_LC_ and *T*_UC_ (see “Materials and methods”) as a way to minimize the identification of nonsense breakpoints. Despite the differences between SAS and Segmented, the two programs were impressively similar in the *T*_LC_s they identified for the six cities studied in Hill et al. (2013) that have been re-analyzed for inclusion in this study. The estimates of *T*_LC_ by SAS and Segmented differed by ≤ 0.6 °C for four of the six cities. For Timmins, whereas SAS estimated 11.9 °C, Segmented estimated 10.2; and for Key West, whereas SAS found no breakpoint, Segmented—true to its tendency to always find something—estimated a value.

Comparing the point-by-point and slope methods we used to extract insulation information from the energy-*T*_a_ plots, the point-by-point method rests on a particularly solid theoretical foundation. It is based directly on the defining equation for calculation of insulation, Eq. (1), which in the present context takes the form *I* = (*T*_LS_ − *T*_a_)/*H*_city_. The method has two limitations: (1) *T*_LS_ is unknown, although it is constrained by normative thermostat-setting behavior (Huchuk et al. 2018), and (2) something must be done to take account of differences in *P* among cities. Analysis of covariance would be the ideal method for taking account of *P* (Packard and Boardman 1988), but—as we learned—the data may fail to meet assumptions of covariance analysis. We calculated the average *P*-normalized city-wide *I* for each city: a procedure entailing the use of *H*_city_/*P*, rather than *H*_city_, in the denominator of Eq. (1). We set *T*_LS_ to three estimated values, and the results of the three resultant analyses are reasonably convergent—pointing to a statistically significant inverse relation between *I* and *T*_10-year_ (Table 3). For future research using the point-by-point method, the most pressing methodological concerns are to better understand strategies for addressing the two limitations.

Population size, *P*, may not be the only useful variable for taking city size into account. A logical alternative would be to use the number of households in the city. The number of households can be estimated as the number of electric utility accounts, and in fact, Hill et al. (2013) used that approach. However, self-reports by electric utilities are the only way of knowing the number of electric accounts, and we have discovered in doing the present research that electric utilities sometimes report implausible numbers of accounts. The US Census Bureau (Lofquist et al. 2012) states that the average number of people per household in the United States is 2.6. We, therefore, expect *P* to be 2.6 times the number of electric accounts. In our full dataset of 42 cities for this report, *P* averages only 2.0 times the number of electric accounts, and part of the reason is that the electric utilities of five cities implausibly reported numbers of accounts close to population size, even when asked a second time. Our present view is that *P* (obtained from government census) is probably, in general, the more reliable measure of city size for purposes of energy analysis.

The slope method for extracting insulation information from energy-*T*_a_ plots is more indirect than the point-by-point method because, rather than being based directly on the definition of insulation [Eq. (1)], it is based on the standard model of city energy use (Fig. 2) (Scholander et al. 1950a; McNab 2002; Hill et al. 2013, 2016). To address the potential confounding effects of *P*, we used a unitless slope method by expressing the slope as a ratio of the calculated energy values at two predetermined *T*_a_s. The use of a unitless slope method has a long history. In many ways, the unitless slope method we use is a direct descendant of the method that Scholander et al. (1950a) employed in their much-cited classic paper that started the modern comparative study of thermoregulatory energetics in mammals. For each species in their study, Scholander et al. expressed metabolic rates at various *T*_a_s as multiples of the species’ RMR or BMR (they used both “resting” and “basal” to refer to this rate), thereby expressing heat production in a unitless way. Scholander et al. then demonstrated the unique usefulness of the unitless approach for comparing species of widely different body sizes: their unitless method allowed them to define major trends in body insulation across species and environments. Our unitless measure, although different from that of Scholander et al., shares some of the same virtues. For our purposes in this paper, the unitless measure has three signal advantages. First, the estimation of *I* as the ratio of two regression-calculated values of *H*_city_ removes the simple, direct effect of city size (*P*), permitting cities of many different sizes to be compared straightforwardly. Second, as a corollary, the ratio remains the same regardless of how *H*_city_ might be normalized for city size; whether city size is measured using *P* or the number of utility accounts, the ratio stays the same. Third, recognizing that the present report is intended to be read by non-specialists in thermoregulatory energetics as well as specialists, the unitless measure *H*_city(-20)_ /*H*_city(10)_ has straightforward meaning for any reader (unlike the units of measure for slope; see Table 3).

A limitation of the unitless slope method is that the *T*_a_s in numerator and denominator must be defined. We chose *T*_a_ = 10 °C in the denominator because 10 °C is a value that, although relatively high, is below the *T*_LC_ of 38 of the 40 cities that have a defined *T*_LC_ (Table 2). We chose *T*_a_ = − 20 °C in the numerator because it is a relatively low but not extreme value, considering North America as a whole. For each city the estimates of *H*_city_ in numerator and denominator were calculated from the city regression equation (Supplementary Table 1). Thus, the calculated ratio reflects all the data available for the city, and a city did not actually need to experience a *T*_a_ as low as − 20 °C for the ratio to be calculated. These are assets of the method. On the other hand, the calculations (by not taking within-city variance directly into account) lose information, and they provide just a single value for each city, thereby reducing the sample size for statistical analysis to equal the number of cities.

### The thermoneutral zone (TNZ)

Hill et al. (2013), in their analysis of six cities, noted a marked tendency for RMR/person (*H*_city_/*P* in the TNZ) to be a function of thermal climate, cold-climate cities having higher size-normalized values than warm-climate cities. Now that we have data on 42 cities (see Supplementary Table 2), it seems clear that that apparent trend was an artifact of the small sample size. We find no evidence for a relationship between RMR/*P* and *T*_10-year_.

In a mammal, the TNZ represents a range of variable insulation. Specifically, the TNZ is a range of *T*_a_ in which body insulation increases as *T*_a_ decreases between *T*_UC_ and *T*_LC_ (Scholander et al. 1950a; McNab 2002; Hill et al. 2013, 2016). The analogy between the TNZ in a city and that in a mammal is complicated and needs to be examined in more detail than heretofore. Here we simply offer brief thoughts on the concept of variable city-wide *I*. Buildings—lacking a mechanism like piloerection (fluffing out of the pelage)—would seem to be far less poised to vary their insulation than a mammal. Nonetheless, two mechanisms exist by which city-wide *I* might, in fact, be variable in the TNZ. First, people in traditional houses do things like close the windows to an increasing extent as *T*_a_ decreases and often ultimately add extra window protection (e.g., storm windows). Second, people vary their personal clothing insulation (e.g. switch tee-shirts for sweaters). One way to think of the TNZ for a city is that it is the range of outdoor temperatures in which people in their dwellings feel no need to use energy-demanding devices to feel comfortable. This formulation helps emphasize that human behavior is an important factor (Rickwood et al. 2008).

SAS and Segmented were impressively similar in the *T*_UC_s they identified for the six cities studied in Hill et al. (2013) that have been re-analyzed for inclusion in this study. Neither program identified a *T*_UC_ for Flagstaff or Timmins. For Ames, Dothan, and Kissimmee, the programs agreed to within 0.5 °C. Whereas SAS estimated the *T*_UC_ of Key West as 21.4 °C, Segmented estimated 22.3 °C.

### Future directions

The premise of this research is that city-wide insulation—an important property for energy analysis—need not be measured in a reductionistic fashion, house by house. Instead, more-efficient methods might be developed to treat the entire city as the unit of measure for quantifying city-wide *I*. Hill et al. (2013) demonstrated that the energy-*T*_a_ plot of an individual city (e.g., Fig. 1B) can be constructed from data routinely gathered by weather stations and energy utilities. The energy-*T*_a_ plot contains information on city-wide insulation. In this paper, we have taken the first steps to develop methods for extracting and applying this insulation information.

We have found that variance is high. Considering any large set of cities, intrinsic differences in relevant city properties undoubtedly contribute to this high variance. Artifacts introduced by the nature of the data available probably also contribute, recognizing that protocols for recording, consolidating, and archiving utility energy data are not highly standardized. The high variance is an impediment to quantifying trends, and for overcoming this impediment, the most obvious next step will be to greatly increase the number of cities investigated.

This study has been self-funded by the authors. As we gathered data from cities, we were in effect acting as private citizens, cold-calling energy utilities to ask for their data. Thus it is not surprising that electric and natural-gas utilities often declined to participate. Hopefully, funding by government agencies will become possible in the future. Utilities might be more willing to provide data when asked under the aegis of a government-funded project, and increased funding would enlarge numbers of personnel available to process the data. In these ways, the number of cities investigated could be increased dramatically, helping to clarify trends despite high variance.

Another important goal that might be realized with more formalized authority and increased personnel would be the analysis of utility substation data within cities to estimate insulation in residential and commercial districts separately. City variance in corporate energy use is undoubtedly a significant contributing factor to city variance in measured insulation. If substation data could be acquired and analyzed separately, it might prove possible to compare cities on the basis of residential districts alone, providing less-confounded, more actionable results.

With the enhancements noted, the study of city-wide *I*—using the city as the unit of measure—might make important contributions to the ongoing project of understanding and managing energy efficiency in the built environment as a potential target of climate-change mitigation (Wilkinson et al. 2007; Rickwood et al. 2008). This research provides the first, tentative insight into how city wide *I* varies with thermal climate in North America, and it suggests more can be done in enhancing *I* to reduce the increased energy usage, and CO_2_ production, incurred for living-space heating in cold climates.

## Supplementary Information

Below is the link to the electronic supplementary material.

Supplementary file1 (DOCX 26 KB)
