# Supplementary material for: City-scale energetics: window on adaptive thermal insulation in North American cities
Source: J Comp Physiol B. 2021 Oct 22;192(1):193–206. doi: 10.1007/s00360-021-01411-8 (PMC8816447; doi:10.1007/s00360-021-01411-8)
Supplement: Supplementary file 1 — Supplementary file1 (DOCX 26 KB) [file 360_2021_1411_MOESM1_ESM.docx]

**Supplementary Information**

**Supplementary Acknowledgments**

**Supplementary Table 1**

**Supplementary Table 2**

**Supplementary Acknowledgments**

The following utilities kindly provided data on daily use of electricity or natural gas: Alabama Municipal Electric Authority (Alexander City, AL; Fairhope, AL), Alexander City Gas Department (Alexander City, AL), Alliant Energy (Ames, IA), AMP Public Power Partners (Danville, VA), Arizona Public Service Co. (Flagstaff, AZ), ATCO Gas (Cardston, Alberta; Fort Macleod, Alberta; Lethbridge, Alberta; Ponoka, Alberta; Red Deer, Alberta), Barrow Utilities Co-op (Utqiagvik, AK), Cedar Falls Utilities (Cedar Falls, IA), Chambersburg Electric Department (Chambersburg, PA), Chambersburg Gas Department (Chambersburg, PA), City of Ames Electric Services (Ames, IA), City of Summerside Electric Utility (Summerside, Prince Edward Island), Danville Utilities (Danville, VA), Dothan Utilities (Dothan, AL), Easton Utilities (Easton, MD), Ellensburg Utility (Ellensburg, WA), ENMAX (Cardston, Alberta; Fort Macleod, Alberta; Ponoka, Alberta; Red Deer, Alberta), Fairhope Public Utilities (Fairhope, AL), Fort Pierce Utilities Authority (Fort Pierce, FL), Gaz Metro (Coaticook, Quebec; Joliette, Quebec; Sherbrooke, Quebec), Harlan Municipal Utilities (Harlan, IA), Henderson Light and Power (Henderson, KY), Henderson Gas (Henderson, KY), Hibbing Public Utilities (Hibbing, MN), Hutchinson Utilities Commission (Hutchinson, MN), Hydro Coaticook (Coaticook, Quebec), Hydro Joliette (Joliette, Quebec), Hydro One (Timmins, Ontario), Hydro-Sherbrooke (Sherbrooke, Quebec), Inuvik Gas Ltd. (Inuvik, Northwest Territories), Keys Energy Services (Key West, FL), Kissimmee Utility Authority (Kissimmee, FL), Lethbridge Electric Utility (Lethbridge, Alberta), Minnesota Power (Hibbing, MN), Municipal Electric Agency of Nebraska (Delta, CO; Glenwood Springs, CO; Holyoke, CO), Municipal Electric Authority of Georgia (Cairo, GA; Lafayette, GA; Lawrenceville, GA; Moultrie, GA; Quitman, GA; Thomasville, GA), Municipal Gas Authority of Georgia (Cairo, GA; Lafayette, GA; Lawrenceville, GA; Moultrie, GA; Quitman, GA; Thomasville, GA), Nephi City Public Works (Nephi, UT), North West Territories Power Corporation (Inuvik, Northwest Territories), Questar Gas (Morgan, UT), Rocky Mount Public Utilities (Rocky Mount, NC), Source Gas (Delta, CO; Glenwood Springs, CO; Holyoke, CO), SEMCO Energy (Baraga, MI), Summit Energy (Blanding, UT), Union Gas (Timmons, Ontario), Unisource Energy Services (Flagstaff, AZ), Utah Associated Municipal Power Systems (Blanding, UT; Morgan, UT), Utah Municipal Power Agency (Nephi, UT), Wisconsin Public Power (Baraga, MI),

**Supplementary Table 1**

**Supplementary Table 1**. Regression results for linear regressions carried out for each city on the daily relationship between *H*_city_ (total rate of city heat production) and average *T*_a_ on all days (see Table 2 for *N*) of the analyzed year when *T*_a_ was lower than *T*_LC_ (see Table 2 for *T*_LC_). Slope and *y*-intercept are presented as mean with SE in parentheses. *H*_city(-20)_ /*H*_city(10)_ is the ratio of predicted *H*_city_ at *T*_a =_ -20°C divided by predicted *H*_city_ at *T*_a =_ 10°C as calculated from the regression. Raw data for Inuvik and Utqiagvik were monthly and weekly, respectively.

| **City** | **Slope  (MW/°C)** | ***y*-intercept (MW)** | ***H*_city(-20)_ / *H*_city(10)_** | **Adjusted r^2^** |
| --- | --- | --- | --- | --- |
| Alexander City, AL | -1.301 (0.1942) | 44.58 (1.517) | 2.24 | 0.27 |
| Ames, IA | -6.589 (0.1481) | 180.4 (1.271) | 2.73 | 0.92 |
| Baraga, MI | -0.327 (0.01003) | 8.646 (0.08699) | 2.82 | 0.81 |
| Blanding, UT | -0.3845 (0.00696) | 9.671 (0.0521) | 2.98 | 0.93 |
| Cairo, GA | -0.7744 (0.2132) | 28.66 (1.52) | 2.11 | 0.25 |
| Cardston, Alberta | -0.7485 (0.0182) | 16.49 (0.1471) | 3.49 | 0.86 |
| Cedar Falls, IA | -5.31 (0.1706) | 151.9 (1.33) | 2.61 | 0.83 |
| Chambersburg, PA | -4.598 (0.1697) | 110.2 (0.9171) | 3.15 | 0.81 |
| Coaticook, Quebec | -0.523 (0.0223) | 20.1 (0.2089) | 2.06 | 0.69 |
| Danville,VA | -11.72 (0.5678) | 355.5 (4.118) | 2.48 | 0.73 |
| Delta, CO | -1.447 (0.06354) | 32.83 (0.3906) | 3.36 | 0.78 |
| Dothan, AL | -6.333 (0.5859) | 188.9 (4.859) | 2.51 | 0.65 |
| Easton, MD | -3.529 (0.1449) | 84.01 (0.9511) | 3.17 | 0.78 |
| Ellensburg, WA | -2.695 (0.05165) | 72.42 (0.392) | 2.78 | 0.92 |
| Fairhope, AL | -2.767 (0.4383) | 72.54 (3.585) | 2.85 | 0.37 |
| Flagstaff, AZ | -15.55 (0.4371) | 376 (2.244) | 3.12 | 0.85 |
| Fort Macleod, Alberta | -0.7052 (0.01462) | 15.46 (0.125) | 3.52 | 0.9 |
| Fort Pierce, FL | -3.431 (1.232) | 114 (12.98) | 2.29 | 0.43 |
| Glenwood Springs, CO | -2.283 (0.07531) | 55.11 (0.5204) | 3.12 | 0.81 |
| Harlan, IA | -0.8337 (0.04061) | 24.77 (0.3156) | 2.52 | 0.66 |
| Hibbing, MN | -1.646 (0.03086) | 44.47 (0.3519) | 2.76 | 0.92 |
| Holyoke, CO | -0.2036 (0.01829) | 8.092 (0.1072) | 2.01 | 0.44 |
| Hutchinson, MN | -2.661 (0.1308) | 95.99 (1.271) | 2.15 | 0.65 |
| Inuvik, NWT | -0.4522 (0.02907) | 11.71 (0.4551) | 2.89 | 0.87 |
| Joliette, Quebec | -3.489 (0.1186) | 151.6 (1.159) | 1.90 | 0.79 |
| Key West, FL | -2.322 (0.376) | 105.6 (5.999) | 1.85 | 0.71 |
| Kissimmee, FL | -12.89 (1.567) | 293.2 (13.35) | 3.35 | 0.79 |
| LaFayette,GA | -0.9226 (0.05994) | 30.69 (0.5236) | 2.29 | 0.55 |
| Lawrenceville,GA | -24.44 (1.017) | 460.9 (7.923) | 4.39 | 0.81 |
| Lethbridge, Alberta | -14.46 (0.3818) | 401.7 (3.324) | 2.69 | 0.87 |
| Morgan,UT | -0.5767 (0.01716) | 11.16 (0.1431) | 4.21 | 0.82 |
| Moultrie, GA | -1.095 (0.2214) | 43.67 (1.948) | 2.00 | 0.24 |
| Nephi, UT | -0.6965 (0.01599) | 26.4 (0.1251) | 2.08 | 0.91 |
| Ponoka, Alberta | -1.249 (0.01849) | 26.92 (0.1844) | 3.60 | 0.95 |
| Quitman, GA | -0.4027 (0.028) | 15.02 (0.3887) | 2.10 | 0.62 |
| Red Deer, Alberta | -14.78 (0.2719) | 350.1 (2.681) | 3.19 | 0.93 |
| Rocky Mount, NC | -9.602 (0.4783) | 260.4 (3.679) | 2.75 | 0.74 |
| Sherbrooke, Quebec | -12.22 (0.4924) | 400.2 (4.22) | 2.32 | 0.74 |
| Summerside, PEI | -0.2447 (0.00936) | 15.15 (0.06647) | 1.58 | 0.77 |
| Thomasville, GA | -2.1 (0.2205) | 105.2 (2.943) | 1.75 | 0.39 |
| Timmins, Ontario | -9.168 (0.2179) | 212.1 (2.449) | 3.28 | 0.88 |
| Utqiagvik, AK | -0.915 (0.02154) | 22.31 (0.3217) | 3.09 | 0.97 |

**Supplementary Table 2**

**Supplementary Table 2**. *T*_UC_, population-normalized RMR, and regression results (slope, *y*-intercept) for linear regression of *H*_city_ (total rate of city heat production) versus *T*_a_ on days when *T*_a_ > *T*_UC_. Values for *N* (number of data points) are specific for each metric. RMR is calculated as mean *H*_city_ per person (SE and *N* in parentheses) for *T*_a_ between *T*_LC_ and *T*_UC_, or for *T*_a_ > *T*_LC_ in cities without defined *T*_UC_. RMR is not calculated for Inuvik or Utqiagvik because of no defined *T*_LC_, nor for Quitman because *T*_LC_ = *T*_UC_. Regression is calculated only for cities with defined *T*_UC_. *ns* = slope of calculated regression not significantly different from zero.

| **City** | ***T*_UC_ (°C)** | **RMR (W/person) (SE, *N*)** | **Regression above *T*_UC_**  **[slope in MW/°C (SE), intercept in MW (SE), *N*]** |
| --- | --- | --- | --- |
| Alexander City, AL | 19.4 | 1410 (33.7, 27) | 0.632 (0.0436), 7.48 (1.06), 155 |
| Ames, IA | 20.6 | 1730 (14.5, 67) | 2.83 (0.528), 45.4 (12.9), 75 |
| Baraga, MI | --- | 1800 (18.8, 82) |  |
| Blanding, UT | 17.2 | 1140 (13.6, 37) | 0.0534 (0.00894), 2.82 (0.206), 120 |
| Cairo, GA | 21.6 | 1890 (23.3, 103) | 0.719 (0.0676), 1.93 (1.75), 135 |
| Cardston, Alberta | --- | 1680 (14.2, 50) |  |
| Cedar Falls, IA | 26.3 | 2090 (23.3, 123) | ns (*N*= 13) |
| Chambersburg, PA | 26.4 | 2560 (27.5, 152) | ns (*N* = 7) |
| Coaticook, Quebec | --- | 1370 (36.6, 74) |  |
| Danville,VA | 21.7 | 4190 (60.1, 66) | 5.84 (1.30), 51.4 (32.8), 92 |
| Delta, CO | 18.4 | 1580 (58.9, 46) | ns (*N* = 105) |
| Dothan, AL | 20.9 | 1660 (13.4, 98) | 5.04 (0.226), 1.22 (6.19), 177 |
| Easton, MD | 19.7 | 2220 (25.4, 53) | 1.58 (0.0774), 2.67 (1.91), 119 |
| Ellensburg, WA | --- | 1710 (13.8, 118) |  |
| Fairhope, AL | 20.3 | 2040 (54.3, 55) | 0.730 (0.0624), 13.3 (1.59), 172 |
| Flagstaff, AZ | --- | 3100 (16.8, 137) |  |
| Fort Macleod, Alberta | --- | 1800 (18.3, 85) |  |
| Fort Pierce, FL | 22.0 | 1700 (16.5, 97) | 2.60 (0.264), 13.6 (6.89), 221 |
| Glenwood Springs, CO | --- | 2210 (28.4, 125) |  |
| Harlan, IA | 19.6 | 2440 (72.1, 25) | ns (*N* = 61) |
| Hibbing, MN | 21.7 | 1340 (12.9, 70) | ns (*N* = 10) |
| Holyoke, CO | 18.9 | 1760 (79.3, 61) | 0.0891 (0.0151), 1.57 (0.356), 96 |
| Hutchinson, MN | --- | 4540 (64.8, 121) |  |
| Inuvik, NWT | --- | --- |  |
| Joliette, Quebec | --- | 4500 (87.0, 73) |  |
| Key West, FL | 22.3 | 2720 (15.9, 48) | 4.66 (0.135), -36.8 (3.74), 277 |
| Kissimmee, FL | 21.6 | 2230 (13.6, 111) | 10.6 (0.213), -95.9 (5.72), 215 |
| LaFayette, GA | 21.1 | 2260 (35.8, 28) | 0.435 (0.134), 6.94 (3.20), 84 |
| Lawrenceville, GA | 19.8 | 3430 (75.0, 61) | 1.07 (0.221), 59.1 (5.39), 140 |
| Lethbridge, Alberta | --- | 2400 (21.4, 77) |  |
| Morgan, UT | --- | 1010 (21.6, 108) |  |
| Moultrie, GA | 21.4 | 1790 (29.7, 45) | 0.942 (0.131), 4.82(3.40), 144 |
| Nephi, UT | 16.4 | 3380 (36.1, 31) | 0.225 (0.0260), 13.6 (0.602), 116 |
| Ponoka, Alberta | --- | 1480 (10.7, 87) |  |
| Quitman, GA | 19.5 | --- | 0.199 (0.0209), 3.30 (0.547), 220 |
| Red Deer, Alberta | --- | 1780 (13.2, 114) |  |
| Rocky Mount, NC | 22.3 | 1910 (35.4, 69) | 4.33 (1.07), 13.3 (27.8), 99 |
| Sherbrooke, Quebec | 19.7 | 1670 (18.5, 74) | ns (*N* = 19) |
| Summerside, PEI | --- | 870 (4.86, 127) |  |
| Thomasville, GA | 22.0 | 3780 (52.5, 40) | 2.14 (0.304), 21.7 (8.10), 145 |
| Timmins, Ontario | --- | 2460 (32.9, 106) |  |
| Utqiagvik, AK | --- | --- |  |
